# Socioeconomic status and breastfeeding in China: an analysis of data from a longitudinal nationwide household survey

**DOI:** 10.1186/s12887-019-1551-2

**Published:** 2019-05-27

**Authors:** Chu Chen, Guo Cheng, Jay Pan

**Affiliations:** 1 West China School of Public Health and West China Fourth Hospital, Sichuan University, No. 16, Section 3, Ren Min Nan Road, Chengdu, 610041 Sichuan China; 2 West China Research Centre for Rural Health Development, Sichuan University, No.17, Section 3, Ren Min Nan Road, Chengdu, 610041 Sichuan China

**Keywords:** Socioeconomic status, Breastfeeding initiation, Breastfeeding duration, Educational status, Occupational status, China

## Abstract

**Background:**

Socioeconomic status is an important factor affecting the initiation and cessation of breastfeeding. However, limited evidence exists regarding the association between socioeconomic status and breastfeeding behavior in China on a national level. This study aims to investigate the relationship between socioeconomic status and the initiation and duration of breastfeeding in China.

**Methods:**

Data were collected from the China Family Panel Studies, a longitudinal nationwide household survey. A total of 2938 infants born between 2010 and 2014 were included in the study. The logistic regression model was used to investigate the relationship between socioeconomic status and the initiation of breastfeeding. Meanwhile, the Cox proportional hazards model was used to investigate the relationship between socioeconomic status and the risk of breastfeeding cessation.

**Results:**

Overall, 90.5% of infants were breastfed, while the average duration of breastfeeding was 8.66 months in China. The breastfeeding continuance rate at 12 months declined sharply, to 30.1%. The study’s findings also indicate that socioeconomic status did not significantly affect breastfeeding initiation. However, infants whose mothers had a high school or higher education and who scored 33–58 on the International Socio-Economic Index of Occupational Status (ISEI) were more likely to experience breastfeeding cessation, as were infants whose fathers had an ISEI score of 59–90.

**Conclusions:**

Efforts to promote breastfeeding practices should be conducted comprehensively to target mothers with a high school or higher education, mothers with a medium occupational status, and fathers with a high occupational status.

## Background

There has been increasing evidence demonstrating the benefits of breastfeeding for both children and mothers. A recent review concluded that breastfeeding was potentially one of the best interventions for reducing mortality in children younger than 5 years of age [1]. Moreover, breastfeeding demonstrated positive long-term effects on childhood obesity, total cholesterol, non-communicable disease occurrences, and intelligence development [2, 3]. For mothers, it can improve birth spacing and reduce the risk of diabetes, ovarian cancer, and breast cancer [4].

Although the health advantages of breastfeeding are well established, the rates of breastfeeding initiation and continued breastfeeding at 2 years, as recommended by the World Health Organization (WHO), are low in most countries [1]. A national survey from the USA reported that 26% of all women, with children aged from 0 to 5 years, did not breastfeed at all [5]. Similarly, in England, 26.1% of mothers did not initiate breastfeeding, and only one third continued breastfeeding at 6 months [6]. Even in Norway, where 98% of mothers initiated breastfeeding, only 35% continued partial breastfeeding for at least a year [7].

China has experienced dramatic economic growth, social polarization, and demographic changes in the past three decades. Its GDP rose from $191,149 billion in 1980 to $11.065 trillion in 2015 [8]. The economic expansion has lifted more than 700 million people out of poverty [9]. Meanwhile, the most salient feature of China’s demographic transformation has been the extensive internal migration from rural to urban areas. Between 2010 and 2015, the number of internal migrants grew from 121 million to 247 million, of which 169 million moved from rural to urban areas [10]. This rapid social and economic transition in China may affect the practice of breastfeeding [11–13]. A review demonstrated that breastfeeding rates in China fell during the 1970s, especially in big cities, and reached their lowest point in the 1980s. In the 1990s, the breastfeeding rate began to grow, with 80% of mothers breastfeeding at 4 months since 1993 [13]. In 2010, a large study conducted in Central and Western China reported that 98.3% of infants had been breastfed, however, only 28.7% children under 6 months were exclusively breastfed, and 55.5 and 9.4% had continued breastfeeding for 1 and 2 years, respectively [14]. In 2013, a breastfeeding initiation rate of 84.6% was reported in the 5th National Health Survey [15]. Although the increasing initiation rate was encouraging, the continued breastfeeding rate at 1 year was still lower than most Asian countries (e.g., Japan, North Korea, and Mongolia) [4], and very few women continued breastfeeding at 2 years or beyond, as recommended by WHO.

Hence, exploring the factors which affect breastfeeding initiation and cessation are crucial for improving the health of mothers and children in China. Previous studies have indicated that socioeconomic status (SES) was an important factor in breastfeeding initiation and duration in China [12, 16–20]. However, this association has not been consistently reported and some studies suggest that mothers with higher educational and occupational statuses were less likely to breastfeed [12, 16–19], while others argued that there was no relationship between the mother’s education or occupation and breastfeeding [14, 20]. Meanwhile, most studies explored the relationship between the mother’s SES and breastfeeding, and seldom considered the father’s SES, which is regarded as a significant factor in determining the initiation and cessation of breastfeeding [21, 22]. Furthermore, there was limited evidence of the association between SES and breastfeeding behavior in China at a national level.

To bridge the gaps in extant literature, this study aims to explore the relationship between SES and the initiation and duration of breastfeeding in China using a nationally representative dataset from a longitudinal household survey. Information from this study will help identify target groups for future breastfeeding promotion projects.

## Methods

### Sample

Data were collected from the China Family Panel Studies (CFPS), funded by China’s Project 985 and conducted by the Institute of Social Science Survey of Peking University. The CFPS was a nationally representative, biennial longitudinal household survey that collected information regarding economic activity, education, and health at the individual, family, and community levels via an interviewer-administered questionnaire. The inaugural survey of the CFPS, conducted in 2010, surveyed a representative sample of 15,000 families and nearly 30,000 individuals within families in 25 provinces or directly governed municipalities in China. The CFPS was conducted according to the guidelines set in the Declaration of Helsinki and all procedures involving human participants were approved by the Ethics Committee of Peking University. Written informed consent was obtained from all subjects [23] (extensive information about the survey can be found at http://www.isss.pku.edu.cn/cfps/en/index.htm).

**Fig. 1:**
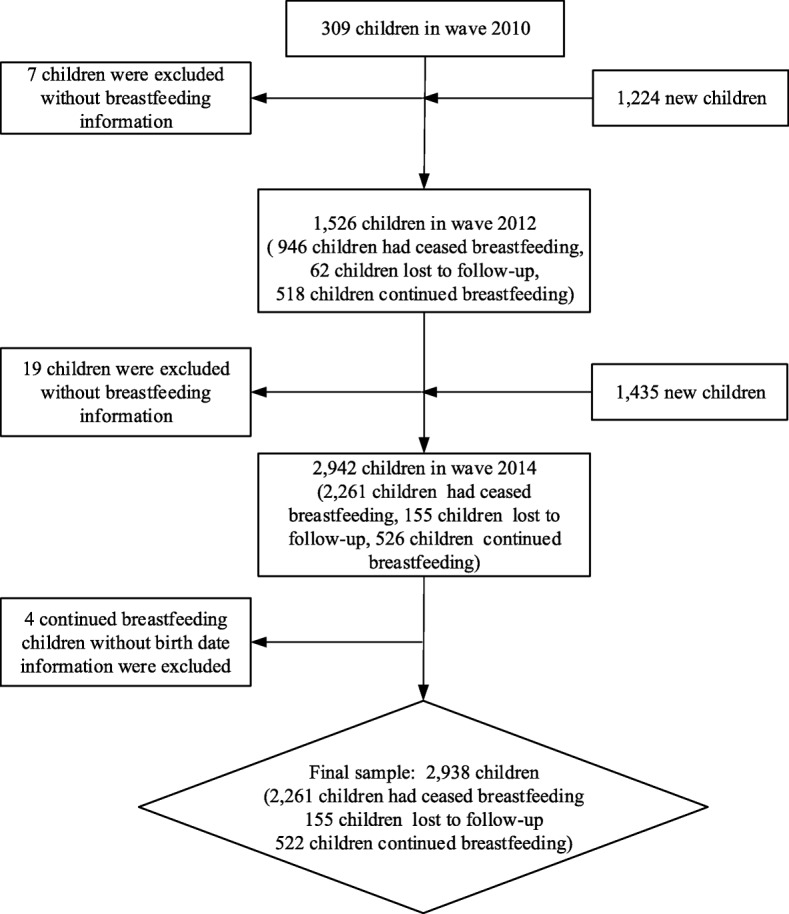
Flow chart of the sample selection process. Notes: Fig. 1 demonstrates the sample selection process. The final sample contains two parts: children who had ceased breastfeeding, which was 2261 (280 infants were never breastfed), and children who had not, namely censored observation. Six hundred seventy-seven children were under censored observation, including 155 lost to follow-up and 522 that continued breastfeeding after the end of the observation time. Thirty children were excluded due to lack of breastfeeding information. Our final sample size was 2938

This study focused on a subgroup of children from the CFPS. Owing to the rapid social and economic development in China, the sample is limited to children born between 2010 and 2014. The initial 2010 CFPS sample comprised 309 infants, while the 2012 and 2014 samples comprised 1526 and 2942 infants, respectively. Thirty children were excluded due to missing breastfeeding information. Excluded samples were compared with the samples used in the analysis. Excepting the father’s occupational and educational status, the mother’s age, and the delivery place of the infant, all other sociodemographic variables (household income per capita, residence, residential region, father’s age, mother’s occupational and educational status, parity, infant’s gender, ethnicity, birth weight, gestational age, and birth year) had no statistical variation between the two groups (*p* < 0.05). Our final sample included 2938 children of which 2261 had ceased breastfeeding (280 infants were never breastfed), 522 had continued breastfeeding, and 155 were lost to follow-up. Figure 1 presents the sample selection process in a flow chart.

### Outcome variables

The breastfeeding initiation and duration data were derived from the following questions: “Is your child still breastfeeding?” Those responding “no” to this question were further asked “For how many months was the child breastfed since he/she was born?” Those responding “yes” in the first question would receive follow-up visits until the mother ceased breastfeeding.

Therefore, the initiation of breastfeeding was defined as instances in which an infant had been breastfed. It was categorized into two groups (non-breastfed, breastfed). As for duration, failure event was defined as breastfeeding cessation. Total survival time was considered the duration of breastfeeding time, expressed in months. As for the children who were still breastfeeding at the last follow-up, the duration was expressed in months from their birth date to the interview date.

### Predictor variables

According to the related literature [24–28], SES was indicated by the household income per capita, parental educational status, and parental occupational status. Household income per capita was positively skewed, thus logarithmic transformation was applied to the income variable. The parental educational status was categorized into two groups (middle school and below, high school and above). The International Socio-Economic Index of Occupational Status (ISEI) score was used to measure parental occupational status. It scores occupations on a continuous scale ranging from 16 to 90, with higher values indicating higher occupational status. The scale is derived from an individual’s education and income level [29–31]. We assigned an ISEI score corresponding to the original scale to each individual occupation in our sample. We then categorized occupational status into four groups (16–32/unemployment, 33–43, 44–58, and 59–90).

### Covariates

The behavior of breastfeeding is associated not only with SES but also with other individual, family, and social factors. As existing literature identifies potentially confounding factors [26, 32–35], we adjusted for the (1) household characteristics: residence (rural, urban), residential regions (Eastern China, Central China, and Western China), (2) parental characteristics: age, marital status of mother (married, single, divorced or widowed) and parity (primipara, multipara), and (3) infant characteristics: gender, birth weight (below 2500 g, normal, above 4000 g), gestational age (less than 37 weeks, 37–42 weeks, and over 42 weeks), place of delivery (health facility, others), ethnicity (Han, minority), and the birth year in our regression model.

Delivery by cesarean section is an important factor affecting breastfeeding behavior in China [36]. However, limited by the CFPS questionnaire, we did not have information regarding the mode of delivery. Therefore, it was not included in this study.

### Statistical analysis

Statistical analyses were conducted using Stata version 14.1. Following descriptive analyses, the logistic regression model was used to analyze the relationship between SES and initiation of breastfeeding. Meanwhile, the Cox proportional hazards model was used to analyze the relationship between SES and the risk of breastfeeding cessation. The 0.05 significance level was used throughout the statistical analysis. The models were set as follows:

1 $$ \mathrm{logit}\ \left[\Pr \left({y}_i=1\right)\right]=\alpha +{\mathbf{SES}}_i\beta +{\mathbf{X}}_i\gamma +{\varepsilon}_i $$

2 $$ {h}_i(t)={h}_0(t)\exp \left({a}_0+{\mathrm{SES}}_i\boldsymbol{\updelta} +{\mathrm{Z}}_i\boldsymbol{\uptheta} +{\mu}_i\right) $$

Equation (1) explores the relationship between SES and the initiation of breastfeeding. Where *i* denotes an individual, *y* denotes whether breastfeeding was initiated. **SES** is a vector, including household income per capita, parental educational status, and parental occupational status. **X** represents a vector of covariates, including (1) household characteristics: residence, residential region, (2) parental characteristics: age, mother’s marital status and parity, and (3) infant characteristics: gender, birth weight, gestational age, place of delivery, ethnicity, and birth year. The error term is denoted by ε. The parameter *β*, the key coefficient of interest, measures the changes of initial breastfeeding on SES. The parameter *γ,* captures the changes of initial breastfeeding on control variables, while *α* is the constant term.

Equation (2) explores the relationship between SES and the duration of breastfeeding. Where *i* denotes an individual, *h(t)* denotes the hazard function, and *h*_*0*_*(t)* denotes the baseline hazard function. **SES** is a vector, including household income per capita, parental education level, and parental occupational status. Vector **Z** contains the same variables as vector **X** in eq. (1). The error term is *μ*. The parameter ***δ***, the key coefficient of interest, measures the changes in the duration of breastfeeding on SES, while the constant term is *α*_*0*_.

## Results

This study included 2938 children, of whom 2658 (90.5%) were breastfed, which was higher than the percentage reported in China’s 5th National Health Survey (84.6%) [15]. As demonstrated in Table 1, the mean duration of breastfeeding was 8.66 months (*SD* = 6.15). The mean household income per capita was RMB 11,482 (*SD* = 28,446). Further, the majority of parents had a low educational and occupational status, lived in rural areas, and were married. Most children were ethnically Han, born at a health facility, and had a normal birth weight. The gender and residential region distribution of children were similar in the study sample.

**Table 1:** Characteristics of sample (*N* = 2938)

Variables	*n* (%)
Household characteristics	
Household income per capita (RMB) (*mean, s.d*)	11,482 (28,446)
Residential regions	
Eastern China	1172 (39.9)
Central China	852 (29.0)
Western China	914 (31.1)
Residence	
Urban	990 (33.7)
Rural	1948 (66.3)
Parental characteristics	
Mother’s educational status	
Middle school and below	2058 (70.1)
High school or above	738 (25.1)
Missing data	142 (4.8)
Father’s educational status	
Middle school and below	1951 (66.4)
High school or above	800 (27.2)
Missing data	187 (6.4)
ISEI score for mother’s occupation	
16–32/unemployment	2079 (70.8)
33–43	480 (16.3)
44–58	256 (8.7)
59–90	123 (4.2)
ISEI score for father’s occupation	
16–32/unemployment	2011 (68.5)
33–43	564 (19.2)
44–58	218 (7.4)
59–90	145 (4.9)
Mother’s marital status	
Single	44 (1.5)
Married	2864 (97.5)
Divorced/widowed	22 (0.7)
Missing data	8 (0.3)
Parity	
Primipara	1653 (56.3)
Multipara	1285 (43.7)
Age of mother (years) (*mean, s.d*)	27.35 (4.79)
Age of father (years) (*mean, s.d*)	29.37 (5.09)
Infant characteristics	
Breastfeeding duration (months) (*mean, s.d*)	8.66 (6.15)
Gender	
Male	1549 (52.7)
Female	1389 (47.3)
Place of delivery	
Health facility	2767 (94.2)
Others	129 (4.4)
Missing data	42 (1.4)
Ethnicity	
Han	2590 (88.2)
Minority	348 (11.8)
Birth weight	
Below 2500 g	126 (4.3)
Normal	2495 (84.9)
Above 4000 g	245 (8.3)
Missing data	72 (2.5)
Gestational age (weeks)	
Less than 37	1490 (50.7)
37–42	1343 (45.7)
Over 42	52 (1.8)
Missing data	53 (1.8)
Birth year	
2010	713 (24.3)
2011	692 (23.6)
2012	648 (22.0)
2013	549 (18.7)
2014	336 (11.4)

The ISEI score refers to occupational status, with higher values indicating higher occupational status

**Fig. 2:**
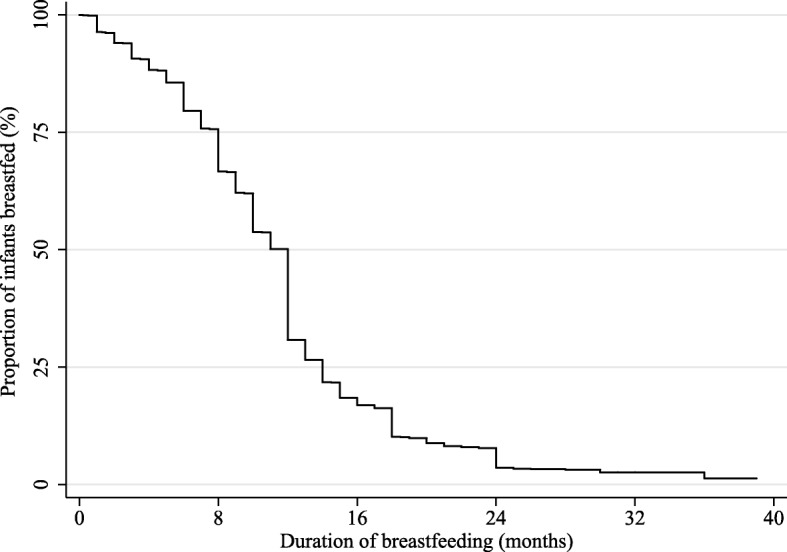
Kaplan-Meier survival curve of breastfeeding duration. Notes: The Kaplan-Meier survival curve of breastfeeding duration indicates that the probability of breastfeeding dropped sharply after 12 months. Overall, the duration of breastfeeding among participants ranges from 0 to 39 months

In Fig. 2, the Kaplan-Meier survival curve of breastfeeding indicates that the probability of breastfeeding dropped sharply after 12 months. Rates of breastfeeding at 6, 12, and 24 months were 79.4, 30.1, and 3.2%, respectively. The duration of breastfeeding among participants ranged from 0 to 39 months.

Table 2 reports the marginal effect of SES on the initiation of breastfeeding, obtained using the logistic regression model. It highlights that SES was not associated with the initiation of breastfeeding.

**Table 2:** The relationship between SES and initiation of breastfeeding (*N* = 2938)

Variables	*AOR* ^*^	95% *CI*	*p*
Household Income per capita (RMB)	0.93	0.82–1.04	0.210
ISEI score for mother’s occupation			
16–32/unemployment (Ref)	1.00		
33–43	0.92	0.60–1.40	0.690
44–58	0.93	0.55–1.57	0.785
59–90	1.09	0.52–2.30	0.816
ISEI score for father’s occupation			
16–32/unemployment (Ref)	1.00		
33–43	0.96	0.66–1.39	0.836
44–58	1.22	0.70–2.11	0.480
59–90	0.61	0.34–1.10	0.103
Mother’s educational status			
Middle school and below (Ref)	1.00		
High school or above	1.10	0.77–1.55	0.608
Father’s educational status			
Middle school and below (Ref)	1.00		
High school or above	1.18	0.86–1.62	0.289

(1) AOR denotes the adjusted odds ratio from logistic regression. The other control variable included: household characteristics (residence, residential regions), parental characteristics (age, mother’s marital status, and parity), and infant characteristics (gender, birth weight, gestational age, place of delivery, ethnicity, and birth year). (2) The ISEI score refers to occupational status, with higher values indicating higher occupational status

Table 3 presents the results from the Cox regression analysis of the relationship between SES and the risk of breastfeeding cessation. We found that mothers with ISEI scores of 33–43 and 44–58 were more likely to cease breastfeeding when compared to those with an ISEI score of 16–32/unemployment by 1.15 and 1.28, respectively. An inverted U-shaped relationship between the occupational status of mothers and breastfeeding duration was presented. Mothers with a high school or higher education, compared with those with an education level of middle school and below, were more likely to cease breastfeeding by 1.14, while infants whose fathers’ ISEI scores were 59–90 were more likely to experience breastfeeding cessation (versus those whose fathers had ISEI scores of 16–32/unemployment) by 1.29.

**Table 3:** The relationship between SES and the risk of breastfeeding cessation (*N* = 2658)

Variables	*AHR* ^*^	95% *CI*	*p*
Household income per capita (RMB)	0.99	0.95–1.03	0.547
ISEI score for mother’s occupation			
16–32/unemployment (Ref)	1.00		
33–43	1.15	1.01–1.32	0.035
44–58	1.28	1.08–1.53	0.005
59–90	1.10	0.86–1.40	0.448
ISEI score for father’s occupation			
16–32/unemployment (Ref)	1.00		
33–43	1.08	0.96–1.22	0.183
44–58	1.02	0.86–1.23	0.793
59–90	1.29	1.04–1.59	0.017
Mother’s educational status			
Middle school and below (Ref)	1.00		
High school or above	1.14	1.01–1.28	0.031
Father’s educational status			
Middle school and below (Ref)	1.00		
High school or above	1.05	0.95–1.18	0.290

(1) AHR denotes the adjusted hazard ratio from Cox regression models. The other control variables included: household characteristics (residence, residential regions), parental characteristics (age, mother’s marital status, and parity), and infant characteristics (gender, birth weight, gestational age, place of delivery, ethnicity, and birth year). (2) ISEI score refers to occupational status, with higher values indicating higher occupational status. (3) 2658 observations were used in Cox regression for 280 infants were never breastfed. Among 2658 children, the longest breastfeeding duration was 39 months, while the shortest was is 0.1 months. The time interval is 0.1 to 39 months

## Discussion

Using data from the CFPS, this study demonstrates that SES does not significantly affect women’s choice to initiate breastfeeding. Infants whose mothers have a high school or higher education and a medium occupational status were more likely to experience breastfeeding cessation, as were infants whose fathers had a high occupational status. Our study makes a unique contribution to the existing literature by using longitudinal data from a nationwide survey of China to explore the relationship between SES and breastfeeding initiation and duration, which would greatly improve the understanding of the relationship between SES and breastfeeding behavior.

The following explanations can be applied to the result of SES not being significantly associated with the initiation of breastfeeding for mothers. First, the Chinese government provides a series of maternity protection schemes for women in both urban and rural areas, possibly reducing the gap between women of different SES levels of breastfeeding awareness. For example, women in both urban and rural areas receive antenatal care service at least five times and postnatal care at least two times, which enriches their knowledge regarding breastfeeding [37]. Second, other factors apart from SES, such as preterm birth, insufficient breast milk, and maternal illness can affect the initiation of breastfeeding, hindering the perceived impact of SES [34, 38, 39].

The results also reveal the relationship between the mother’s SES and the duration of breastfeeding. In general, our findings are consistent with previous studies in developing countries, which report that a higher educational and occupational status of the mother could result in a shorter duration of breastfeeding [17, 40]. However, we only found that infants whose mothers had an ISEI score of 33–58 (e.g., shop, stall, and market salespersons and demonstrators, waitresses, and bartenders) were more likely to experience breastfeeding cessation. This suggests that mothers with a medium occupational status were more likely to stop breastfeeding. A partial explanation for this result may lie in maternity employment causing the cessation of breastfeeding.

Owing to economic growth, social polarization, and demographic changes, in 2013, 346.4 million women in China were employed, of which 28.98% had a high school education or above [41, 42]. However, short maternity leaves and lack of accommodation for mothers to express milk in their workplace are an obstacle in continuing breastfeeding. In China, employed women receive a 98-day paid maternity leave, which may be extended by 15 days under special circumstances such as birth complications [43]. Women must return to work after their 3–3.5 months of maternity leave. However, only 2.6% of the workplaces in China have breastfeeding rooms [13], which may impact the mothers’ decision regarding continuing breastfeeding. Another possible reason may be that inappropriate marketing for milk formula influences the mothers’ decision to continue with breastfeeding. While research reports negative health consequences associated with formula use, many women may believe that infant formula is better and more convenient than breastfeeding due to advertising [44]. Therefore, women, especially those with a medium SES [45], are more likely to choose milk formula after returning to work. Mothers with a high occupational status may have better working conditions and better knowledge regarding milk formulas, which may result in continued breastfeeding.

Overall, our findings indicate the importance of the father’s role in breastfeeding duration, which is consistent with previous findings that fathers greatly influence mothers in prolonging breastfeeding [22, 46, 47]. We found that infants of fathers with high occupational status (ISEI score of 59–90, e.g., directors, chief executives, and engineers) experienced shorter breastfeeding duration. Lack of paid paternity leave and emotional support for their partner may be the reasons for this. In China, there was no paid paternity leave for fathers before 2017 [48]. Additionally, fathers with high occupational status may be busier and seldom provide emotional support for their partner. Thus, the father’s role in breastfeeding should be enhanced.

The limitation of this study is that some potential confounding factors could not be controlled for in analyses due to the available data. Future research should consider including more factors, e.g., delivery mode (cesarean section or vaginal delivery), maternal and infant illness, and grandmother’s attitudes towards and prior experience with breastfeeding.

## Conclusion

The breastfeeding initiation rate in our study was higher than that reported in the 5th National Health Survey; however it demonstrates a sharp decline in continued breastfeeding at 12 months. Further, the results suggest that SES does not significantly impact women’s choice to initiate breastfeeding. Infants whose mothers have a high school or higher education and a medium occupational status (e.g., shop, stall, and market salespersons and demonstrators, waitresses, and bartenders) were more likely to experience breastfeeding cessation, as were infants whose fathers had a high occupational status (e.g., directors, chief executives, and engineers). Efforts to promote breastfeeding practices should be conducted comprehensively to target mothers with a high school or higher education and a medium occupational status and fathers with high occupational status. Moreover, breastfeeding accommodation at work should be provided, while the milk formula market should be regulated.

## Data Availability

The datasets used during the current study are available in the Institute of Social Science Survey, it can be found at http://www.isss.pku.edu.cn/cfps/en/index.htm.

## Abbreviations

AHR: Adjusted Hazard Ratio
AOR: Adjusted Odds Ratio
CFPS: China Family Panel Studies
CI: Confidence Interval
ISEI: International Socio-Economic Index of Occupational Status
SES: Socioeconomic Status
WHO: World Health Organization
